# AGING IN PLACE IN A MARGINALIZED COMMUNITY AT RISK OF RURAL DEPOPULATION

**DOI:** 10.1093/geroni/igae098.1331

**Published:** 2024-12-31

**Authors:** Yuichi Watanabe

**Affiliations:** Musashino University, Nishitokyo, Tokyo, Japan

## Abstract

Japan is known as one of the most aged societies globally. As of 2022, the aging ratio is approximately 29%. According to the Ministry of Internal Affairs and Communications, in 2019, over 50% of the population in 20,000 neighborhoods, known as “marginalized communities,” are 65 or older. These communities are at a higher risk of rural depopulation or local extinction. Between 2009 and 2019, questionnaire surveys were conducted every two years to assess the experiences and strengths of residents in a marginalized community. The aim was to identify ways to promote sustainable living in the community. As each survey sample size was small, the analysis used all samples as a pooled dataset (N=831). The questionnaire included variables related to people’s anticipation of permanent community living and their engagement in “IBASHO” activities. In Japan, “IBASHO” refers to a place where people can feel secure, have roles, and consider their lives meaningful. A logistic regression analysis was conducted to determine the relationship between anticipation of permanent community living and engagement in “IBASHO” activities. The study revealed that residents who participate in “IBASHO” activities are more likely to feel at ease living in their community permanently, even if they live alone or have poor health. These individuals care for one another in their “IBASHO” community, sharing their strengths and anxieties and providing emotional and practical support. Encouraging “IBASHO” activities can help promote permanent community living, even in areas with an aging population.

